# Hypocalcaemia in patients with prostate cancer treated with a bisphosphonate or denosumab: prevention supports treatment completion

**DOI:** 10.1186/s12894-018-0393-9

**Published:** 2018-09-20

**Authors:** Jean-Jacques Body, Roger von Moos, Daniela Niepel, Bertrand Tombal

**Affiliations:** 1 Department of Medicine, CHU Brugmann, Université Libre de Bruxelles, Place A.Van Gehuchten 4, 1020 Brussels, Belgium; 2 Kantonsspital Graubünden, Loëstrasse 170, CH-7000 Chur, Switzerland; 3 Global Medical Affairs, Amgen (Europe) GmbH, Zug, Switzerland; 4 Institute of Clinical Research, Université Catholique de Louvain, Avenue Mounier 50, 1200 Brussels, Belgium

**Keywords:** Bone-targeted agents, Bisphosphonate, Denosumab, Zoledronic acid, Prostate cancer, Hypocalcaemia

## Abstract

**Background:**

Most patients with advanced prostate cancer develop bone metastases, which often result in painful and debilitating skeletal-related events. Inhibitors of bone resorption, such as bisphosphonates and denosumab, can each reduce the incidence of skeletal-related events and delay the progression of bone pain. However, these agents are associated with an increased risk of hypocalcaemia, which, although often mild and transient, can be serious and life-threatening. Here we provide practical advice on managing the risk of hypocalcaemia in patients with advanced prostate cancer who are receiving treatment with bone resorption inhibitors. Relevant references for this review were identified through searches of PubMed with the search terms ‘prostate cancer’, ‘bone-targeted agents’, ‘anti-resorptive agents’, ‘bisphosphonates’, ‘zoledronic acid’, ‘denosumab’, ‘hypocalcaemia’, and ‘hypocalcemia’. Additional references were suggested by the authors.

**Main text:**

Among patients with advanced cancer receiving a bisphosphonate or denosumab, hypocalcaemia occurs most frequently in those with prostate cancer, although it can occur in patients with any tumour type. Consistent with its greater ability to inhibit bone resorption, denosumab has shown superiority in the prevention of skeletal-related events in patients with bone metastases from solid tumours. Consequently, denosumab is more likely to induce hypocalcaemia than the bisphosphonates. Likewise, various bisphosphonates have differing potencies for the inhibition of bone resorption, and thus the risk of hypocalcaemia varies between different bisphosphonates. Other risk factors for the development of hypocalcaemia include the presence of osteoblastic metastases, vitamin D deficiency, and renal insufficiency. Hypocalcaemia can lead to treatment interruption, but it is both preventable and manageable. Serum calcium concentrations should be measured, and any pre-existing hypocalcaemia should be corrected, before starting treatment with inhibitors of bone resorption. Once treatment has started, concomitant administration of calcium and vitamin D supplements is essential. Calcium concentrations should be monitored during treatment with bisphosphonates or denosumab, particularly in patients at high risk of hypocalcaemia. If hypocalcaemia is diagnosed, patients should receive treatment with calcium and vitamin D.

**Conclusion:**

With preventative strategies and treatment, patients with prostate cancer who are at risk of, or who develop, hypocalcaemia should be able to continue to benefit from treatment with bisphosphonates or denosumab.

## Background

Bone metastases are common in patients with prostate cancer and may develop in up to 90% of patients with advanced disease [1]. Skeletal-related events (SREs; including pathologic fracture, spinal cord compression, radiation to bone, and surgery to bone) are serious complications of bone metastases, are associated with increased pain, morbidity, and mortality, and therefore negatively affect patient quality of life [2]. Although bone metastases in patients with prostate cancer are frequently of the bone-forming osteoblastic type, biochemical and histological analyses suggest that there is also an excess of osteoclast activity in these lesions, leading to bone destruction and an increased risk of SREs [3]. Indeed, analysis of bone metastases type in 1487 patients with prostate cancer showed that 76.5%, 4.8%, and 18.7% of patients had osteoblastic, osteolytic, or mixed bone metastases, respectively (Amgen, data on file).

Inhibitors of bone resorption, such as the bisphosphonate zoledronic acid and the fully human monoclonal antibody against the receptor activator of nuclear factor kappa B ligand (RANKL) denosumab, reduce the incidence of SREs in patients with prostate cancer and bone metastases [3, 4]. Zoledronic acid and denosumab are indicated for the prevention of SREs in adults with advanced malignancies involving bone [5–7]. Therefore, the labels include prostate cancer patients with bone metastases irrespective of whether their disease is hormone-sensitive or castration-resistant. The development of bone metastases is a key event in the progression of castration-resistant prostate cancer and both agents can be used in men with this disease and bone metastases to prevent SREs [8]. The clinical effectiveness of these therapies in patients with castration-sensitive prostate cancer is yet to be demonstrated [9]. If not treated with an inhibitor of bone resorption, almost half of patients with prostate cancer and bone metastases could develop a SRE [4]. Denosumab is a more efficacious inhibitor of bone resorption than zoledronic acid and has shown superiority in the prevention of SREs [3, 10]. Both agents have been associated with an increased risk of hypocalcaemia, but the risk is greater with denosumab than with zoledronic acid, consistent with the greater efficacy of denosumab to inhibit bone resorption and reduce skeletal morbidity [3, 11]. Hypocalcaemia may lead to discontinuation of denosumab treatment; in a retrospective real-world study of 104 patients with bone metastases from solid tumours who were receiving denosumab, four patients discontinued treatment because of hypocalcaemia [12].

Patients with prostate cancer and osteoblastic metastases who are receiving treatment with inhibitors of bone resorption are at a particularly high risk of hypocalcaemia [11]. Although often mild and transient, hypocalcaemia can also present as a serious and life-threatening condition, which can lead to treatment interruption or cessation. However, with proper patient monitoring, hypocalcaemia can be prevented and treated. In this review, we provide practical advice on managing the risk of hypocalcaemia in patients with advanced prostate cancer who are receiving treatment with bone resorption inhibitors. Relevant references for this review were identified through a literature search of PubMed (limited to English-language publications) with the search terms ‘prostate cancer’, ‘bone-targeted agents’, ‘anti-resorptive agents’, ‘bisphosphonates’, ‘zoledronic acid’, ‘denosumab’, ‘hypocalcaemia’, and ‘hypocalcemia’. Additional references relevant to the topics of focus in the review were suggested by the authors based on their expert knowledge of the therapy area.

## Main text

### Diagnosing hypocalcaemia

Calcium homeostasis is mediated by the effect of active vitamin D (calcitriol) and parathyroid hormone (PTH) on the gastrointestinal (GI) absorption of calcium, renal excretion of calcium, and osteoclast/osteoblast activity in the skeleton (which is the main calcium sink in the body) [13]. Consequently, hypocalcaemia has many potential causes, such as vitamin D deficiency (which can lead to secondary hyperparathyroidism), abnormal magnesium or phosphate levels, and partial or complete hypoparathyroidism [14, 15]. Hypocalcaemia can range in severity from mild asymptomatic cases to acute life-threatening crises [16]; the Common Terminology Criteria for Adverse Events (CTCAE) define grades of hypocalcaemia from mild (grade 1) to severe (grade 5) (Table 1) [17].

**Table 1:** Common Terminology Criteria for Adverse Events grading of hypocalcaemia [17]

Grade	Total corrected calcium concentration, mmol/l (mg/dl)
1	2.0–2.1 (8.0–LLN)
2	1.75 to < 2.0 (7.0 to < 8.0)
3	1.5 to < 1.75 (6.0 to < 7.0)
4	< 1.5 (< 6.0)
5	If death occurs as a result of hypocalcaemia

*LLN* lower limit of normal

Extracellular calcium is required for the normal functioning of muscles and nerves. Thus, signs and symptoms of hypocalcaemia include muscle twitching, spasms, tingling, and numbness, and patients with severe hypocalcaemia may develop tetany, seizures, and cardiac dysrhythmias [18]. The development of neuromuscular excitability depends on both the absolute concentration of calcium and how rapidly the concentration has fallen. Patients who experience a rapid fall in serum calcium concentration are often symptomatic, whereas those who develop hypocalcaemia gradually may be asymptomatic, with hypocalcaemia being diagnosed as an incidental biochemical finding [18]. However, if asymptomatic hypocalcaemia is not treated, long-term consequences can include neuropsychiatric symptoms, cataract formation, and raised intracranial pressure [18]. Such cases predominantly occur in patients with chronic hypoparathyroidism [19].

### Measuring calcium concentrations

Approximately 50% of serum calcium exists in an unbound ionized form, and 50% is bound to protein (predominantly albumin) [18]. Only unbound ionized calcium is physiologically active; therefore, serum calcium concentration must be corrected for albumin concentration in order to confirm a diagnosis of hypocalcaemia [16]. This is particularly important in patients with advanced cancer in whom albumin levels are frequently low [20]. Hypocalcaemia is defined as a corrected serum total calcium concentration below 2.1 mmol/l (ionized calcium < 1.1 mmol/l) [21]. Corrected calcium can be calculated using the following formula: measured total calcium (mmol/l) + 0.02 × [40 – measured albumin level (g/l)] [22]. Analysis of unbound ionized calcium concentration provides the most accurate measurement of serum calcium and is recommended in patients who are seriously ill [16]: unfortunately, ionized calcium measurements are not often routinely available. Analysis of creatinine, phosphate, magnesium, vitamin D, and PTH levels is also recommended when diagnosing hypocalcaemia in order to identify other underlying causes of low serum calcium concentrations [16].

### Identifying patients who are at high risk of hypocalcaemia

It is rare for patients with cancer to develop hypocalcaemia as a direct result of the primary tumour [23]. However, low calcium concentrations are quite frequently observed in individuals with osteoblastic bone metastases, in whom rapid mineralization of newly formed bone sequesters calcium from the bloodstream [24, 25]. In retrospective analyses, conducted before the introduction of bisphosphonate or denosumab treatment, hypocalcaemia was reported in 5–13% of patients with bone metastases, depending on the formula used to correct for albumin levels. Among these patients, 35% had hypocalcaemia of grade 1, 60% grade 2, and 5% grade 3 or higher [25]. Prevalence was highest among individuals with prostate cancer and bone metastases; 13–27% of these patients developed hypocalcaemia [25].

**Fig. 1:**
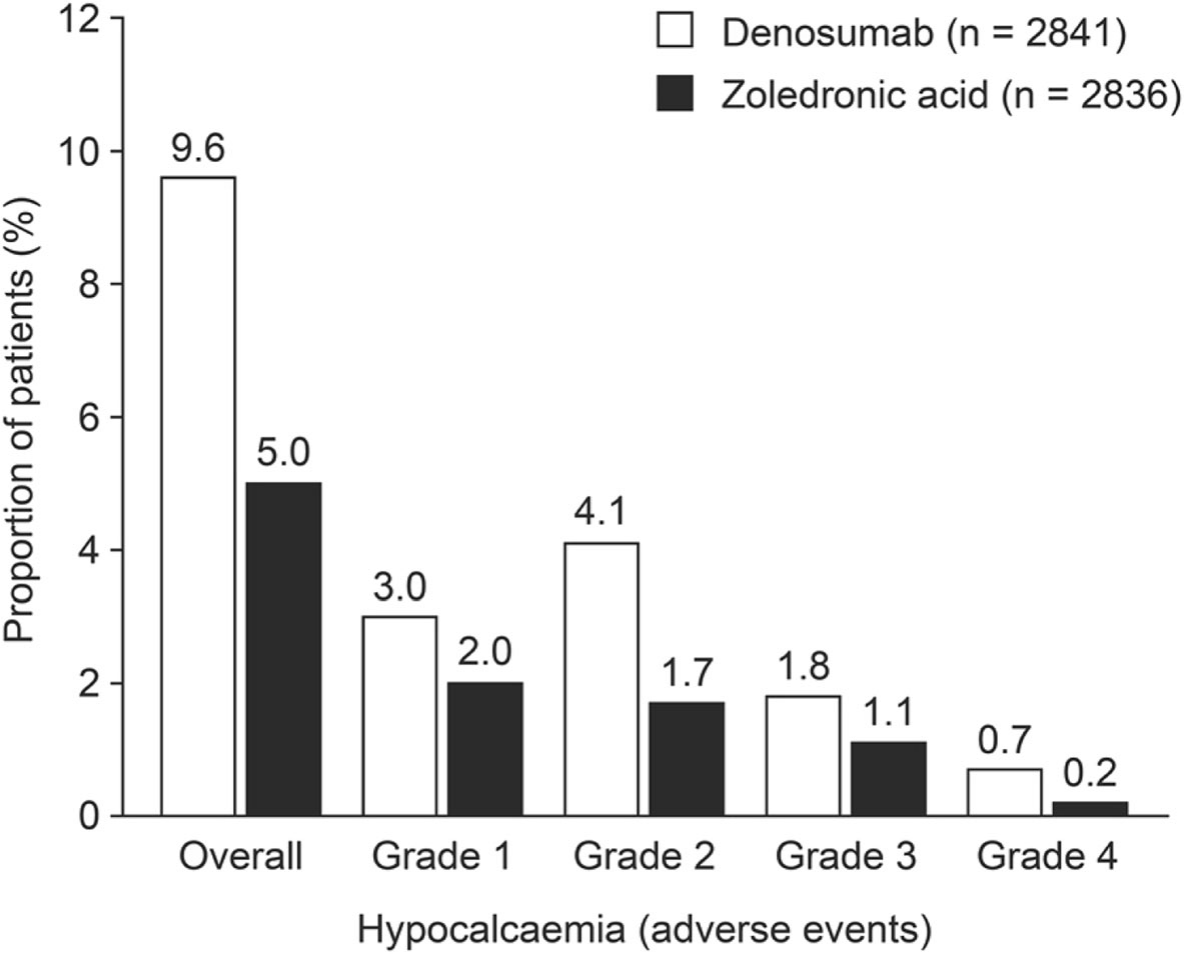
Proportion of patients receiving denosumab or zoledronic acid who developed hypocalcaemia of any grade. Figure reproduced with permission from Body et al. Eur J Cancer 2015;51:1812–21 under the Creative Commons licence (https://creativecommons.org/licenses/by-nc-nd/4.0/) [11]

Hypocalcaemia has been reported as an adverse event associated with the use of bisphosphonates (such as zoledronic acid) and denosumab. Bisphosphonates are analogues of a natural regulator of bone metabolism, pyrophosphonate. They localise to the extracellular bone matrix from where they may prevent osteoclast differentiation, induce osteoblasts to produce osteoclast inhibitory factors, and cause apoptosis of osteoclasts [26]. Denosumab is a monoclonal antibody against RANKL that disrupts signalling through RANK, thus preventing tumour-mediated activation of osteoclasts [27]. Bisphosphonates and denosumab lead to reduced osteoclast activity, thus reducing bone resorption and the release of calcium from bone into the bloodstream [11]. Preclinical and clinical data indicate that denosumab is a more efficacious inhibitor of osteoclast activity than zoledronic acid [11] and has shown superiority in the prevention of SREs compared with zoledronic acid [3, 10]. Consistent with the potent action of denosumab, in a combined analysis of three clinical trials comparing denosumab and zoledronic acid in patients with bone metastases from solid tumours (including prostate cancer) or multiple myeloma, hypocalcaemia of any grade occurred more frequently with denosumab than with zoledronic acid (9.6% vs. 5%, respectively) (Fig. 1) [11].

Real-world data suggest that the incidence of hypocalcaemia associated with bisphosphonates or denosumab may be higher than has been reported in clinical trials. Retrospective studies of patients treated with denosumab have reported an incidence of 9–22% for hypocalcaemia of grade 2 or higher [21, 28–30]. A 12-month observational study of 125 patients with bone metastases (including 92 patients with prostate cancer) who were receiving zoledronic acid showed that hypocalcaemia of any grade occurred in 18.5% of the patients with prostate cancer. Of patients with any tumour type, hypocalcaemia of grade 3 or 4 occurred in 4% [31]. The higher incidence of hypocalcaemia encountered in real-world studies compared with clinical trials may reflect poor adherence to guidelines for calcium supplementation and monitoring of patients in clinical practice [12]. Although both clinical trial data and real-world evidence suggest that severe hypocalcaemia is a relatively rare occurrence, it can be a serious, life-threatening condition that requires hospitalization and administration of intravenous calcium [28]. However, hospital admissions due to hypocalcaemia associated with inhibitors of bone resorption remain low, suggesting that such cases are often mild [30].

Analysis of data from phase III clinical trials has identified several factors that increase the risk of hypocalcaemia [11]. Hypocalcaemia associated with inhibitors of bone resorption has been shown to occur in patients with bone metastases from a variety of primary tumour types, but is most frequently seen in those with prostate cancer or small-cell lung cancer [11]. Additionally, male sex and the presence of osteoblastic metastases have been shown to associate significantly with an increased risk of hypocalcaemia [11]. By inhibiting bone resorption and preventing the release of calcium from bone, bisphosphonates and denosumab further increase the bone mineralization and calcium sequestration associated with osteoblastic metastases [32].

Hypocalcaemia in patients with cancer frequently relates to a poor nutritional status, and these individuals often have low albumin and/or vitamin D concentrations [24]; therefore, as discussed above, it is important to correct serum calcium measurements for albumin levels. Moreover, vitamin D deficiency is associated with an increased risk of hypocalcaemia following treatment with inhibitors of bone resorption [33, 34] and is common in elderly people and those with cancer [35, 36]. Hence, vitamin D deficiency is a concern in men with prostate cancer, given that their median age at diagnosis is 66 years [37]. Indeed, more than 40% of men have been shown to be deficient in vitamin D (serum calcidiol < 20 ng/ml) at the time of diagnosis of prostate cancer [38].

Patients with severe renal insufficiency (glomerular filtration rate < 30 ml/min) or stage 4 or 5 chronic kidney disease (CKD) have an increased risk of hypocalcaemia associated with treatment involving inhibitors of bone resorption [21, 29]. This increased risk of hypocalcaemia likely results from CKD-induced secondary hyperparathyroidism [21]. Additionally, CKD causes a reduction in the activity of 1-α-hydroxylase and conversion of vitamin D to the active form (calcitriol), resulting in the reduced intestinal absorption of calcium [39]. Denosumab is a useful therapeutic option for patients with advanced cancer and renal insufficiency or CKD because, in contrast to zoledronic acid, it is not excreted by the kidneys and requires no dose adjustment for renal insufficiency [6, 40]. However, owing to the high risk of hypocalcaemia in patients with severe renal insufficiency, we advise caution when considering such patients for treatment with denosumab, and if treatment is administered, such patients should be closely monitored [29].

Patients with high levels of bone turnover markers (i.e. urinary N-telopeptide of type I collagen and bone-specific alkaline phosphatase) at baseline are also at an increased risk of hypocalcaemia [11]. Additionally, patients with pre-existing hypocalcaemia, associated with impaired parathyroid function, have a high risk of developing hypocalcaemia following treatment with inhibitors of bone resorption [41].

### Preventing hypocalcaemia

**Fig. 2:**
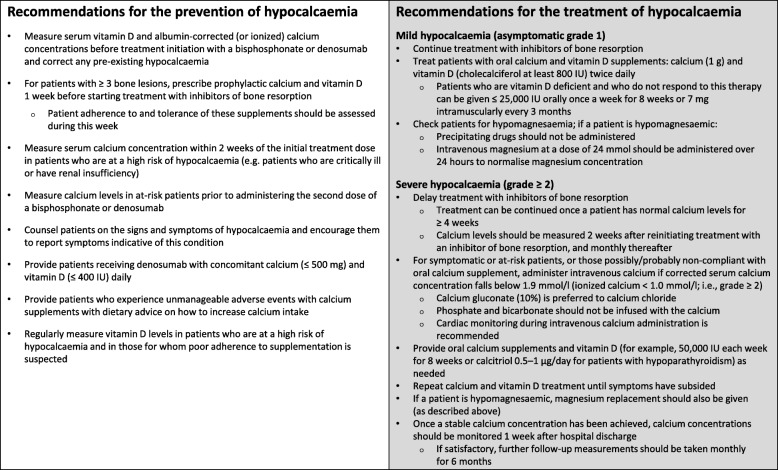
Recommendations for the prevention and treatment of hypocalcaemia

Hypocalcaemia associated with inhibitors of bone resorption is avoidable in most cases (Fig. 2). With proper management, patients should be able to continue to benefit from treatment with these agents, significantly reducing the risk of developing SREs, which may be painful or associated with increased mortality [2].

**Fig. 3:**
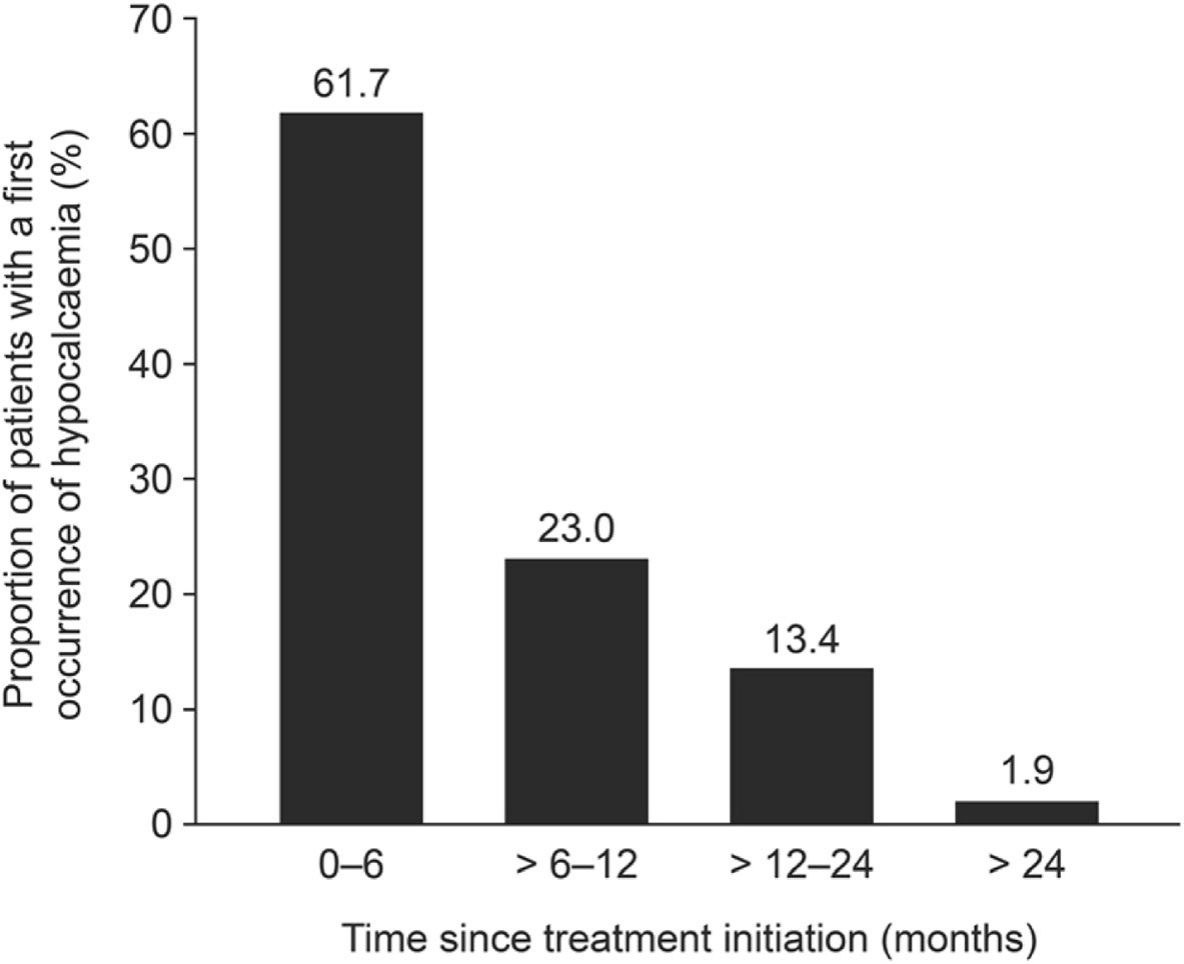
First occurrence of hypocalcaemia by time period in patients receiving denosumab (*n* = 313) [11]

Although hypocalcaemia can occur at any time during therapy, it is most frequently reported within 6 months of treatment initiation (Fig. 3) and occurs earlier in patients receiving denosumab than in those receiving zoledronic acid; in phase III trials, the median time to hypocalcaemia of grade 2 or higher was 3.8 months with denosumab and 6.5 months with zoledronic acid [11]. Similar findings have been reported in real-world studies; a retrospective chart review of 55 patients with advanced cancer who were receiving denosumab found that, in most patients, hypocalcaemia developed shortly after treatment administration (median 16 days) and after a median of one injection (range 1–14) [28]. Another retrospective study, conducted in 66 patients with cancer who received a median of 3–6 cycles of denosumab, found that the incidence of hypocalcaemia of any grade was higher during the first course of therapy (16.7%) than in second or later courses (6.1%) [29]. However, long-term clinical trial safety data on denosumab in patients with bone metastases from breast cancer or prostate cancer showed that the incidence of hypocalcaemia did not increase with longer exposure to denosumab [42].

To prevent hypocalcaemia in patients receiving a bisphosphonate or denosumab, serum vitamin D and albumin-corrected (or ionized) calcium concentrations should be measured before treatment initiation. Pre-existing hypocalcaemia must be corrected before treatment initiation [5, 6]. For patients with a substantial tumour burden in the bone (≥ 3 bone lesions), prophylactic calcium and vitamin D should be prescribed 1 week before starting treatment with a bisphosphonate or denosumab. During this week, patient adherence to and tolerance of these supplements should be assessed. The denosumab label recommends that serum calcium concentration should be measured within 2 weeks of the initial dose in all patients and that additional monitoring should be conducted in patients with suspected symptoms of hypocalcaemia and in those at high risk of hypocalcaemia [6]. However, in clinical practice, we recommend such monitoring following the initial dose of a bisphosphonate or denosumab only in patients who are at a high risk of hypocalcaemia, such as those who are critically ill or who have renal insufficiency. Nevertheless, we do recommend measuring calcium levels in at-risk patients [11] prior to administering the second dose of a bisphosphonate or denosumab. Additionally, patients should be counselled on the signs and symptoms of hypocalcaemia and encouraged to report symptoms indicative of this condition [6]. Monitoring allows early detection of hypocalcaemia and hence the correction of serum calcium concentration before administering further doses of a bisphosphonate or denosumab.

### Calcium and vitamin D supplements

**Fig. 4:**
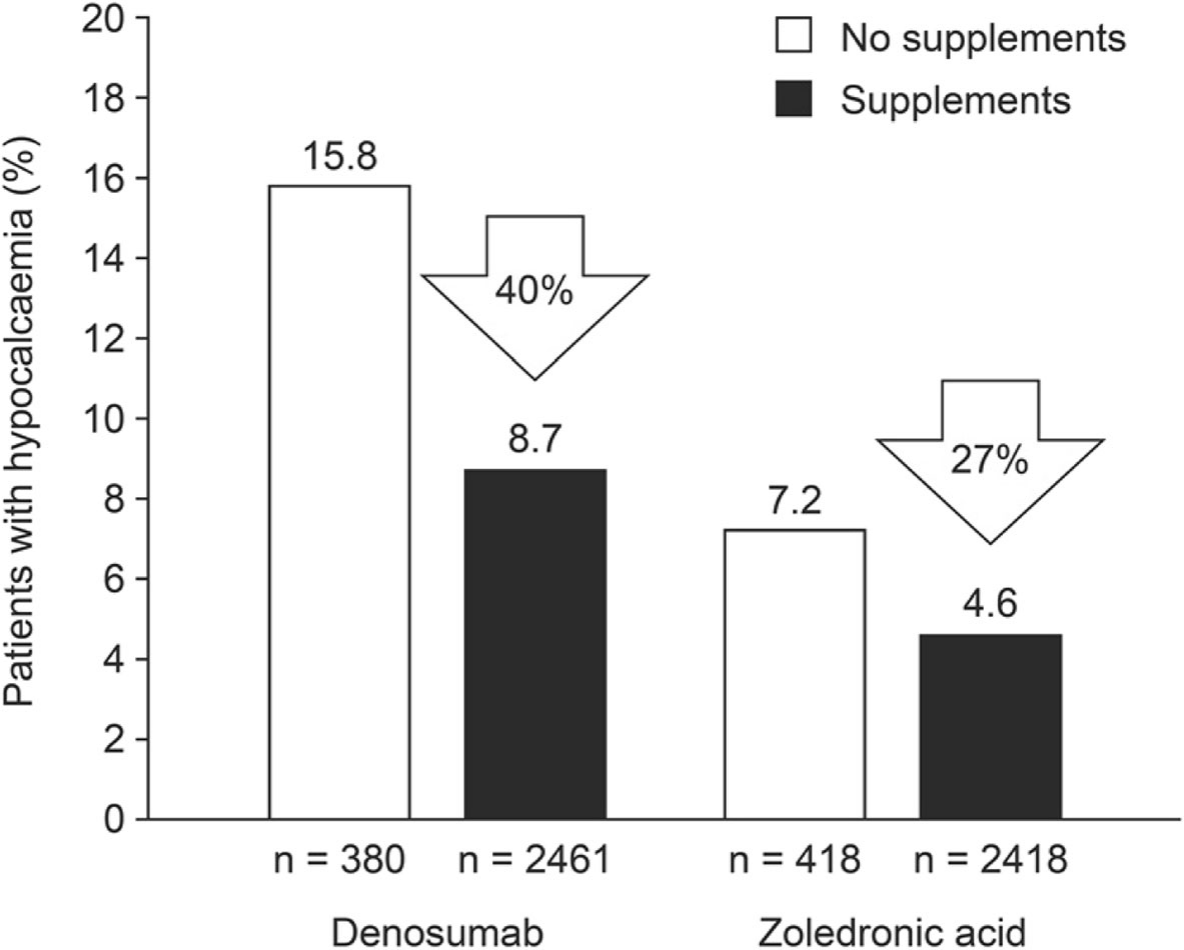
Hypocalcaemia risk in patients receiving calcium and/or vitamin supplementation versus those who were not [11]

Preventative supplementation with calcium and/or vitamin D is associated with fewer hypocalcaemia adverse events for patients receiving zoledronic acid or denosumab. Use of these supplements [recommended doses: calcium ≥ 500 mg/day; vitamin D ≥ 400 International Units (IU) /day] has been shown to lower the risk of developing hypocalcaemia by 27% or 40% in patients receiving zoledronic acid or denosumab, respectively (Fig. 4) [11]. This reduction in risk is impressive given that, in this analysis, patients were considered to be receiving supplements if they had reported taking oral calcium and/or vitamin D at any time during the study (excluding those who reported supplement use only after their first hypocalcaemia event) [11].

Adequate supplementation is necessary in all patients treated with a bisphosphonate or denosumab [5, 6]. For patients receiving denosumab, concomitant supplementation with a minimum of 500 mg calcium daily and 400 IU vitamin D daily is required [6]. When managing hypocalcaemia risk in patients with bone metastases from prostate cancer, it is important to note that those receiving androgen-deprivation therapy should also receive calcium and vitamin D supplements as part of the preventative treatment for cancer treatment-induced bone loss [43].

The recommended daily intake of calcium varies by guideline and by patient population. To avoid calcium deficiency, the British Dietetic Association recommends that adults consume 700 mg of calcium daily [44]. The National Institutes of Health recommends a daily intake of calcium of 1000 mg for adults aged up to 70 years (1200 mg for women aged 51–70 years) and 1200 mg for those aged over 70 years [45]. Elderly patients have an increased requirement for calcium due to age-associated reduction in intestinal calcium absorption [46]. For all patients, the percentage of calcium absorbed depends on the total amount of elemental calcium consumed at one time. The percentage absorption decreases as the amount of calcium increases: absorption is maximal when doses of 500 mg or less are consumed at one time [45].

The two main forms of calcium in supplements are carbonate and citrate [47, 48]. Calcium carbonate should be taken with food because absorption is dependent on stomach acid, whereas calcium citrate is absorbed equally well when taken with or without food and is the preferred form for patients receiving proton pump inhibitors [48]. Other calcium forms in supplements include calcium gluconate, calcium chloride, calcium lactate, calcium phosphate, and calcium citrate malate [45, 46].

The main circulating form of vitamin D is calcidiol [49]. Vitamin D also exists in two inactive forms, namely vitamin D_2_ (ergocalciferol) and vitamin D_3_ (cholecalciferol), which are hydroxylated in the liver to form calcidiol and then in the kidneys to form the biologically active form, calcitriol [49]. Vitamin D deficiency is defined as a serum calcidiol concentration below 20 ng/ml (50 nmol/l), and insufficiency is defined as a calcidiol level of 21–29 ng/ml (52–72 nmol/l) [49]. Patients with a calcidiol concentration below 10 ng/ml (25 nmol/l) can be considered to be severely deficient [50]. Vitamin D deficiency can be caused by reduced synthesis via the skin (owing to limited sun exposure, skin pigmentation or skin thinning as a result of ageing), decreased vitamin D absorption, and increased catabolism [16, 18, 49]. Calcitriol enhances intestinal absorption of calcium and promotes bone mineralization and remodelling [16]: there is a significant decrease in intestinal calcium absorption when serum vitamin D levels fall below 30 ng/ml [49].

Similar to calcium daily reference values, the recommended daily intake of vitamin D varies by guidelines and depends on the patient population being treated. The amount of vitamin D supplementation required to achieve adequate serum levels (> 30 ng/ml or > 75 nmol/l) depends on a variety of factors in addition to the baseline vitamin D levels including age, body mass index, sun-exposure history, and the use of medications that can affect vitamin D metabolism and intestinal absorption [51]. Among patients who are at risk of vitamin D deficiency, the Endocrine Society Clinical Practice Guidelines recommend that adults aged up to 70 years receive a preventative dose of vitamin D 600 IU daily, and those older than 70 years receive 800 IU daily [52]. The International Osteoporosis Foundation recommends that adults aged 60 years and over receive 800–1000 IU daily [53].

Many treatment regimens are available for patients with vitamin D deficiency. The Endocrine Society Clinical Practice Guidelines recommend that these patients should be treated with 50,000 IU of ergocalciferol or cholecalciferol weekly for 8 weeks or 6000 IU daily until a serum calcidiol level above 75 nmol/l is achieved. This should be followed with maintenance therapy of 1500–2000 IU daily [52]. NICE recommends that fixed loading doses of vitamin D (up to a total of 300,000 IU) be given as either weekly or daily split doses, followed by lifelong maintenance treatment of 800 IU daily [54]. Higher doses of up to 2000 IU daily, and occasionally much higher doses, may be used for certain groups of people (e.g., those with malabsorption disorders) [54]. Other treatment regimens include 50,000 IU once a week for 6 weeks (300,000 IU in total), 20,000 IU twice a week for 7 weeks (280,000 IU in total), or 4000 IU daily for 10 weeks (280,000 IU in total) [54]. For the treatment of vitamin D insufficiency, some guidelines recommend that maintenance doses should be started without the use of loading doses; however, we strongly recommend the use of vitamin D loading doses in patients who are deficient before starting therapy with an inhibitor of bone resorption [54].

Vitamin D can be given as ergocalciferol or cholecalciferol, or as the active metabolites alfacalcidol or calcitriol [55]. Ergocalciferol and cholecalciferol are both recommended for the treatment and prevention of vitamin D deficiency [52], although cholecalciferol is the more potent of the two agents [56] and is thus prescribed more commonly than ergocalciferol. The normal conversion of vitamin D to its active form is compromised in patients with severe renal insufficiency (glomerular filtration rate < 30 ml/min); therefore, calcitriol or alfacalcidol should be administered for the treatment of hypocalcaemia in these patients [57]. Treatment with calcitriol or alfacalcidol can lead to rapid changes in serum calcium levels, so patients should be monitored closely in order to avoid hypercalcaemia [58].

Adherence to supplements, particularly calcium supplements, is often low, which can have a negative impact on efficacy [47]. In patients receiving a bisphosphonate or denosumab, measures should be taken to maximise adherence to calcium and vitamin D supplements: the risk of hypocalcaemia and the importance of adherence to supplements should be discussed with patients, and serum calcium levels should be monitored to assess adherence. Regular patient follow-up to encourage treatment compliance may help to ensure that supplements are taken correctly.

Compliance may be enhanced by choosing a suitable dose and delivery method for calcium and vitamin D supplements. Calcium and vitamin D supplements are available as separate or combined formulations, as a variety of delivery compounds including oral tablets, capsules, soft-chews, powders, or as solution [46, 47]. Calcium carbonate, available as powder formulations, has been shown to have increased bioavailability compared with calcium citrate, most often given as tablet formulations [47]. However, patient preference studies of calcium and vitamin D supplements have shown that soft-chew tablets are preferred over powder formulations [59]. In addition, patients are more likely to be adherent with once-daily dietary supplements than with twice-daily supplements [60]. However, the advantages of once-daily supplementation depend on the dose required and should be considered in light of the reduced percentage absorption of calcium when it is given in doses above 500 mg [45]. As discussed above, vitamin D supplements can be given weekly or biweekly [54], which may improve adherence compared with daily supplements.

Calcium supplements are associated with a number of adverse events that may reduce patient compliance with treatment [61]. In particular, gastrointestinal (GI) adverse events are common in patients taking calcium supplements [61]. Compared with calcium citrate, calcium carbonate is more often associated with GI symptoms [48]. Although most GI adverse events are mild, a meta-analysis assessing the safety of calcium supplements in randomised trials found a significantly increased incidence of hospital admissions for GI adverse events in patients receiving calcium (6.8%) when compared with those receiving placebo (3.6%) [62]. For patients who experience unmanageable adverse events with calcium supplements, it is essential to provide dietary advice on how to increase calcium intake with calcium-rich foods (Table 2) [44, 63]. In contrast, vitamin D supplements are generally well tolerated [64], but we advise regular monitoring of vitamin D levels in patients who are at a high risk of hypocalcaemia and those for whom poor adherence to supplementation is suspected.

**Table 2:** Foods rich in calcium [44, 63]

Food type	Quantity	Calcium (mg)
Dairy sources of calcium		
Cow’s milk (all types)	200 ml	240
Sheep’s milk	200 ml	380
Hard cheese (e.g., cheddar, parmesan, emmental)	30 g	240
Fresh cheese (e.g., cottage cheese, ricotta, mascarpone)	200 g	138
Soft cheese (e.g., camembert, brie)	60 g	240
Feta	60 g	270
Mozzarella	60 g	242
Cream cheese	30 g	180
Yoghurt	120 g	200
Calcium-enriched fromage frais	50 g	125
Calcium-enriched low-fat spread	28 g	121
Malted milk drink	25 g serving in 200 ml milk	440–710
Hot chocolate (light)	25 g serving in 200 ml water	200
Rice pudding	200 g	176
Custard	120 ml	120
Non-dairy sources of calcium		
Sardines (with bones)	60 g	258
Pilchards (with bones)	60 g	150
Whitebait	50 g	130
White beans	80 g raw/200 g cooked	132
Wholemeal bread	100 g	100
Non-dairy calcium-fortified products		
Calcium-enriched milk alternatives (e.g., rice, soya, oat, nut, coconut)	200 ml	240
Soya bean curd/tofu^a^	60 g	200
Calcium-enriched orange juice	250 ml	195
Calcium-fortified cereals	30 g	137
Calcium-fortified bread	40 g	191

^a^Only if set with calcium chloride (E509) or calcium sulphate (E516)

### Treating hypocalcaemia

#### Mild hypocalcaemia

Patients with mild hypocalcaemia (asymptomatic grade 1) should be treated with oral calcium and vitamin D supplements: calcium (1 g) and vitamin D (cholecalciferol at least 800 IU) should be given twice daily [65]. Patients who are vitamin D deficient and who do not respond to this therapy can be given higher doses of vitamin D: at least 25,000 IU orally once a week for 8 weeks or, where available and if needed, 7 mg intramuscularly every 3 months [65]. It is important to check whether a patient is hypomagnesaemic, which induces functional hypoparathyroidism leading to low serum calcium levels [66]. If a patient is hypomagnesaemic, precipitating drugs should not be administered. To normalise magnesium concentration, intravenous magnesium at a dose of 24 mmol should be administered over 24 h [67]. We recommend that treatment with inhibitors of bone resorption be continued in patients who experience grade 1 hypocalcaemia.

#### Severe hypocalcaemia

For symptomatic or at-risk patients [11], or those possibly/probably non-compliant with oral calcium supplement, intravenous calcium should be administered if corrected serum calcium concentration falls below 1.9 mmol/l (ionized calcium < 1.0 mmol/l; i.e., grade ≥ 2) [16]. Asymptomatic patients with a corrected serum calcium below 1.9 mmol/l can rapidly become critically ill, and so it is important to treat and monitor these patients closely [18].

Intravenously administered calcium gluconate (10%) is preferred to calcium chloride, which is a strong irritant. It can be given as repeated 50–100 ml boluses in 5% dextrose or by continuous infusion [16, 67]. If calcium chloride is given, it should be administered via a central line to prevent irritation of veins [67]. In order to avoid precipitation of calcium salts, phosphate and bicarbonate should not be infused with the calcium [16]. Patients with severe hypocalcaemia should also receive oral calcium supplements (as described above) and vitamin D (e.g., 50,000 IU each week for 8 weeks or calcitriol 0.5–1 μg/day for patients with hypoparathyroidism) as needed [18]. Treatment should be repeated until symptoms have subsided. It should be noted that hypocalcaemia can be prolonged, so continuous administration of a dilute solution of calcium for a few days may be required [18, 68]. Magnesium replacement should also be given (as described above) to those who are hypomagnesaemic [18, 67]. Cardiac monitoring during intravenous calcium administration is recommended because the rapid elevation of serum calcium concentration required to correct hypocalcaemia can result in cardiac arrhythmia [18].

Retrospective analysis of patients treated for denosumab-associated hypocalcaemia found that the median time from detection of hypocalcaemia to normocalcaemia was 33 days (range: 9–92 days) [28]. However, the time to normalization of serum calcium levels depends on the baseline calcium concentration, the treatment regimen, and how frequently calcium levels are measured. Once a stable calcium concentration has been achieved, calcium concentrations should be monitored 1 week after hospital discharge. If satisfactory, further follow-up measurements should be taken monthly for 6 months [18, 67]. We recommend that treatment with inhibitors of bone resorption be delayed in patients who experience grade 2 or higher hypocalcaemia; treatment can be continued once a patient has had a stable normal calcium level for a minimum of 4 weeks. In patients who have experienced severe hypocalcaemia, serum calcium levels should be measured 2 weeks after reinitiating treatment with an inhibitor of bone resorption, and monthly thereafter. Our recommendations for the treatment of hypocalcaemia are summarised in Fig. 2.

## Conclusion

Hypocalcaemia associated with the use of bisphosphonates and denosumab can be serious; however, it is both preventable and treatable. Given the reduction in skeletal morbidity and the potential improvements to quality of life that inhibitors of bone resorption offer patients with cancer and bone metastases, potential barriers to their use should be properly understood and addressed. With appropriate patient monitoring and preventative measures, patients with advanced prostate cancer, including those with additional risk factors for hypocalcaemia, should be able to continue to benefit from treatment with these agents.

## Abbreviations

CKD: Chronic kidney disease
CTCAE: Common Terminology Criteria for Adverse Events
GI: Gastrointestinal
PTH: Parathyroid hormone
RANKL: Receptor activator of nuclear factor kappa B ligand
SREs: Skeletal-related events
